# Candida auris infection; diagnosis, and resistance mechanism using high-throughput sequencing technology: a case report and literature review

**DOI:** 10.3389/fcimb.2023.1211626

**Published:** 2023-12-08

**Authors:** He Hong, Yang Ximing, Ma Jinghan, Abdullah Al-danakh, Pan Shujuan, Lin Ying, Yang Yuting, Liu Yuehong, Yao Xingwei

**Affiliations:** ^1^ Department of Clinical Laboratory, Dongzhimen Hospital of Beijing University of Chinese Medicine, Beijing, China; ^2^ Department of Urology, The First Affiliated Hospital of Dalian Medical University, Dalian, China

**Keywords:** *Candida auris* infection, drug resistance, gene mutation, epidemiology and prevention, virulence

## Abstract

**Background:**

*Candida auris* (*C. auris*), a recently developing fungal disease with high virulence, easy transmission, and substantial medication resistance in hospitals, poses a growing danger to human health. In 2009, the initial documentation of this disease was made when it was discovered in the ear canal of an elderly Japanese patient. Since its initial isolation, the presence of *C. auris* across six continents has been a cause for severe concern among medical professionals and scientists. According to recent findings, *C. auris* is connected with five geographically different lineages and significant rates of antifungal resistance. Furthermore, *C. auris* infections in healthcare settings lack appropriate treatment options and standardized strategies for prevention and control. This results in many treatment failures and hinders the elimination of *C. auris* in healthcare institutions. To examine the drug resistance mechanism of *C. auris* and to aid in clinical therapy, we provide a case of *C. auris* infection along with a short review of the relevant literature.

**Clinical presentation:**

An 81-year-old female with cerebral hemorrhage was admitted to the hospital and diagnosed with a urinary catheter-related *C. auris*. The sample was evaluated and reported in terms of culture, identification, drug sensitivity, and gene sequencing. We also evaluated the relationship between the morphology of the isolated strains and their drug resistance. Whole-genome sequencing yielded the genes ERG11-Y132F, CDR1-E709D, TAC1B-Q503E, and TAC1B-A583S; however, no additional loci included alterations of concern, according to our results. ERG11-Y132F and TAC1B-A583S are drug-resistant gene loci, whereas CDR1-E709D and TAC1B-Q503E are unidentified variants.

**Conclusion:**

We discover a *C. auris* case of specific a strain in an old female that has some drug-resistant genes, and some genes may be different from already reported gene sites. Gene locus, mutation, and drug resistance mechanism studies may contribute to the creation of innovative drugs and therapeutic treatments. Clinicians and microbiologists must be aware of this globally spreading yeast, which poses substantial hospital diagnostic, treatment, and infection control challenges. Future multicenter research must be performed to uncover this health threat and provide new, effective treatments.

## 1 Introduction

*C. auris* is a recently discovered multidrug-resistant yeast pathogen that is infamous for generating nosocomial outbreaks in a broad variety of healthcare settings all over the globe. Critically ill individuals have been shown to have significantly higher rates of *C. auris* infection-related death (Spivak and Hanson, 2018). Patients of any age who have been hospitalized for an extended period of time in an intensive care unit (ICU), have had prior contact with antibiotics with a wide spectrum of activity, and are undergoing extensive medical interventions are at an increased risk of contracting *C. auris* (Calvo et al., 2016). When the pathogen was subjected to biochemical-based identification methods using commercial systems such as Vitek 2, API ID32C, and Auxacolor, the yeast was initially misidentified as other yeast species. These other yeast species included *Candida haemulonii, Candida famata, Candida sake, Rhodotorula glutinis, and Saccharomyces cerevisia*e. Therefore, *C. auris* was initially misidentified as other yeast species (Rossato and Colombo, 2018). Internal transcribed spacer (ITS) and 28S ribosomal DNA (rDNA) gene sequencing approaches, as well as matrix-assisted laser desorption ionization-time of flight spectrometry systems (MALDITOF MS), have allowed for more accurate identification and differentiation of *C. auris* compared to other *Candida species* (Calvo et al., 2016). Studies conducted on hospital monitoring demonstrated that this opportunistic infection might be acquired through dry and wet environmental surfaces in healthcare settings (Piedrahita et al., 2017). The development of numerous virulence factors, such as hydrolytic enzymes, and the capacity to form biofilms that assist in the persistent colonization of *C. auris* on human skin and the environmental surface have been linked with the ability to survive for an extended period of time on hospital surfaces (Piedrahita et al., 2017). As a result, the use of disinfectants and antiseptics that are of a higher level of efficacy is urgently required in order to improve hospital infection control procedures (Fu et al., 2020).

Even though *C. auris* has garnered a lot of attention in the clinical and scientific communities, and even though a lot of research and review articles on the topic have been published over the past few years, our knowledge of the organism’s biology and virulence characteristics is still quite limited. This research analyzes a strain of *C. auris* that was received from the microbiology laboratory at our hospital. This investigation provides new information on the culture morphology, identification, and drug resistance genes of *C. auris*.

## 2 Case presentation and discussion

### 2.1 Patient history, investigation, and diagnosis

An 81-year-old female patient with more than ten months of consciousness disturbance and limb paralysis was treated with anti-infection, anti-hypertension, hypoglycemic, and thromboprophylaxis treatments. The patient was in a coma, so she was admitted to the hospital with a diagnosis of “cerebral hemorrhage,” in which endotracheal intubation for invasive breathing, a stomach tube, and a urinary catheter were inserted. She had a fever and was receiving broad-spectrum antibiotics without improvement, so we performed a normal urine examination that revealed white blood cells (WBC) 3+, WBC 253/ul, followed by a urine culture study.

*Candida albicans* was identified in the first urine culture. The patient’s temperature rose, so she was treated with symptomatic cooling and fluconazole, as an antifungal medication. She continues to report urinary symptoms, so they did for her urine culture. Two weeks later, the urine culture revealed *C. auris* with a colony count greater than 105 CFU/ml. Based on the results of the urine culture, the previously used antifungal treatment effect of fluconazole was not effective, so oral voriconazole was initiated. One week later, the blood test results were reviewed: WBC: 10.7*109/L, RBC: 2.60*1012/L, NE%: 83.1%, CRP: 52.37mg/L, PCT: 0.07ng/ml, and urine test: LEU: 3+. The WBC count was 236/ul, and the core body temperature fluctuated around 38.8°C. Based on the treatment effect and drug sensitivity findings, voriconazole resistance was noted and it was discontinued and itraconazole capsules were administered. The remaining symptomatic treatments were identical to those previously used. a week later, the patient’s body temperature fell to 36.4°C, but the urine culture continued to reveal *C. auris.* The patient continued to experience urinary tract infection symptoms despite taking antifungal medication, but her condition improved and she was discharged from the hospital with regular follow-up visits.

**Figure 1:**
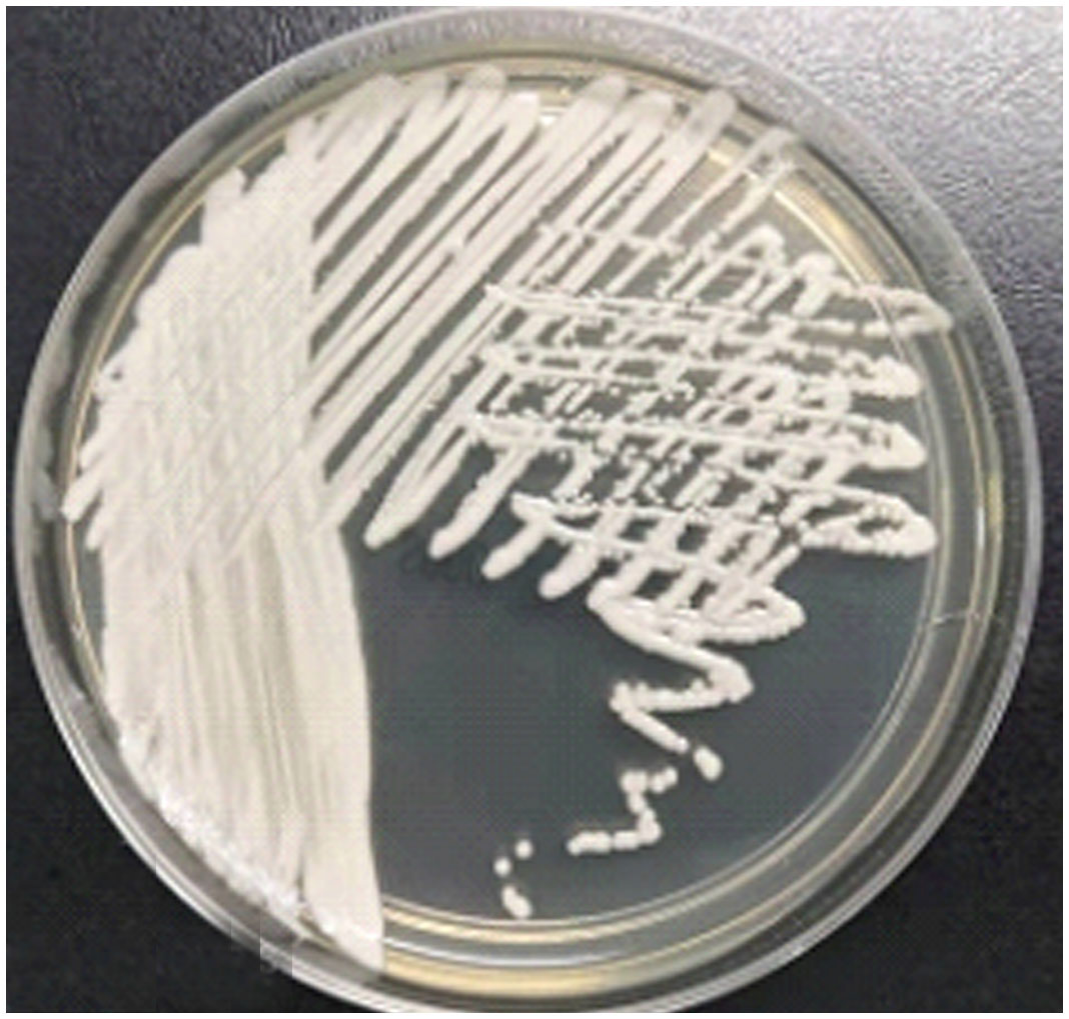
48-hour colony morphology on Sabouraud plates.

In compliance with the national standard operating procedures for clinical examination, the microbiological laboratory inoculated the provided urine samples quantitatively. With quantitative inoculation rings, Columbia blood agar, Mackay agar, and sapaul agar were inoculated and cultivated in 35°C CO2 and 28°C incubators. After 24 hours on Columbia blood agar and sapaul agar, >10^5^ tiny colonies had formed. After 48 hours, white colonies appeared on the blood plate, and the larger colony growing on the sapaul plate was milky white (**Figure 1**), similar to other common fungal colonies. Similar to the previous literature, it indicates that the color-developing plate is white at 24 hours and gradually develops pink at 48 hours (Ong et al., 2019). After several MALDI - TOF MS (antof ms1000 of Antu Biology) identifications, with a score of 9.0, it was confirmed to be *C. auris*(**Supplementary Figure 1**), and the species identification results were reliable.

**Figure 2:**
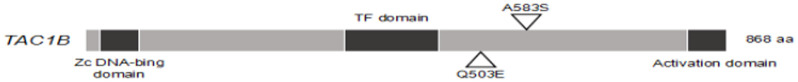
TAC1B transcriptional gene variation sites, of which the gray part represents the azole resistance gene.

Splicing was performed using WA (software for sequence alignment with a small difference to a large reference gene), bqsr (base quality calibration pair) &calling (resequencing), bcftools (tool for detecting mutations and processing), filtering, snpeff (genome structure data annotation software) annotation, genome *de novo* (*de novo* sequencing), and spades (data software) analysis. Whole genome sequencing (WGS) can not only identify strains but also detect related genes of strains. The genome is about 13M, and N50 (the length of genome splicing is half of the total length) is 30K, 3314 SNPs (nucleotide polymorphisms), and indels (insertion deletions) were analyzed. This investigation identified amino acid site variation at ERG11-Y132F, CDR1-E709D, transcription factor variation TAC1b-Q503E, and TAC1b-A583S (**Figure 2**), but no suspicious mutation was found in other sites.

After that, the antifungal drug susceptibility test using the colorimetric microdilution method was conducted that determine the sensitivity of *C. auris* to common antifungal drugs. The results revealed that 5-fluorocytosine (FC) was sensitive, but the second drug sensitivity breakpoint for Amphotericin B (AB) was not identified, and NCCLS M27 suggested that minimum inhibitory concentration (MIC) ≥ 2 was recommended to be determined as drug resistance, ITR was sensitive, and Voriconazole (VOR) was resistance (**Table 1** shows detailed MIC for each all tested drugs with their effectiveness). Similarly, the trial MIC breakpoints recommended by the Centers for Disease Control and Prevention (CDC) in the United States, which define resistance as (AB 2 mg/l drug resistance, fluconazole (FZ) 32 mg/l drug resistance, and no other azole medications) (Arendrup et al., 2017; Chaturvedi et al., 2008).

**Table 1:** Antifungal drugs susceptibility test results using the colorimetric microdilution method.

Antifungal drugs	MIC (ug/mL)	Result	Drug demarcation point range				
			S	Susceptible-dose dependent (S-DD)	I	R	Non-sensitive
AND	0.12	S	≤2				>2
MF	0.12	S	≤2				>2
CAS	0.12	S	≤2				>2
FC	0.06	S	≤4		8-16	≥32	
PZ	0.06	S	≤1		2	≥4	
VOR	8	R	≤1	2		≥4	
IZ	0.12	S	≤0.125	0.25-0.5		≥1	
FZ	64	R	≤8	16-32		≥64	
AB	2	R	≤1			>1	

AND, anidulafungin; MF, micafengin; CAS, caspofungin; FC5-fluorocytosine, PZ, posaconazole; VOR, voriconazole; IZ, itraconazole; FZ, fluconazole; AB, amphotericin; B, MIC minimal inhibitory concentration; S, sensitive; R, resistance; I, intermediate.

### 2.2 Case study discussion

The isolation of *C. auris* from the urine of a hospitalized woman in our institute in China is described in this study. Although most previously known *C. auris* strains showed multidrug resistance, Therefore, whether the drug resistance of previously reported *C. auris* isolates is a recently evolved feature is unknown. FZ and VOR were not used, and Triazole antifungal medications were not advised based on the drug sensitivity results. Despite the fact that IZ was present, clinical effectiveness was poor. Echinocandin concentrations in urine were low, and therapy was usually ineffective. Therefore, it was recommended that AB be used with FC and IZ for clinical treatment.

**Figure 3:**
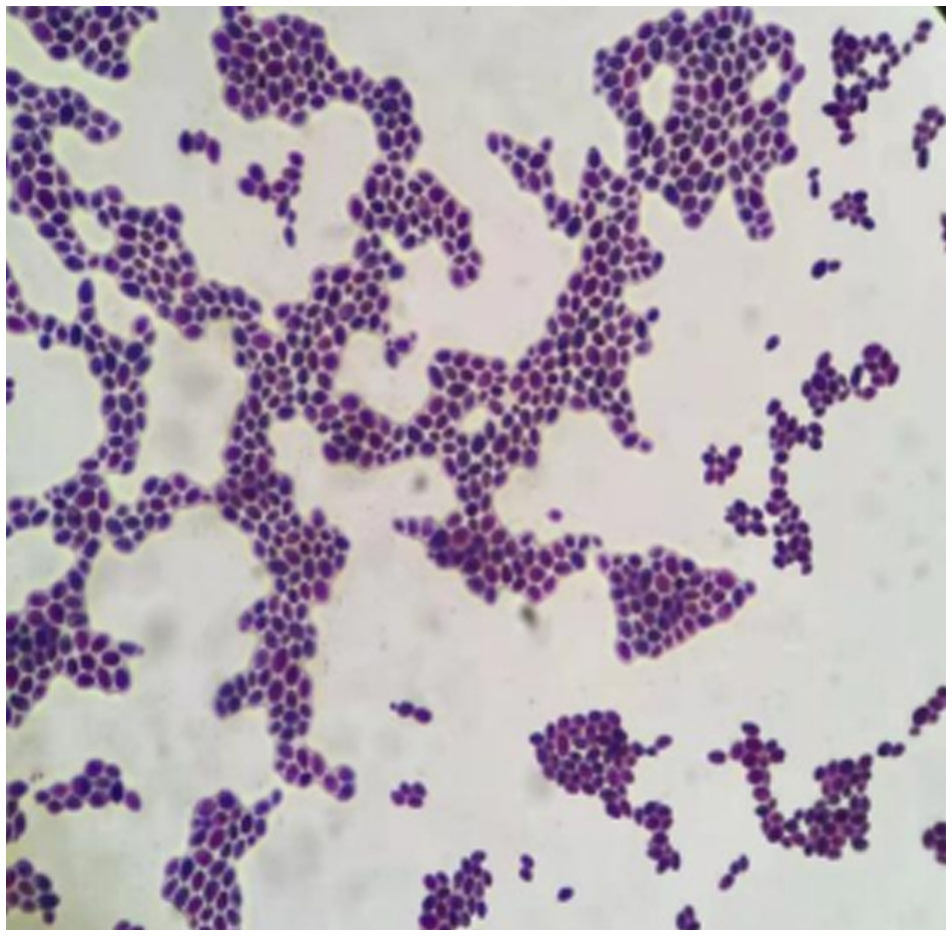
Morphology of *Candida auris* gram stained under 40x microscope.

*C. auris* has aggregated, non-aggregated, and mycelial forms (Guiqiang, 2020), and isolated strains prefer to aggregate when examined under the microscope (**Figure 3**). Aggregated *C. auris* is more resistant to AB than non-aggregated candida, implying that aggregated candida is more resistant to AB, which is consistent with the drug sensitivity data in our patient (Ben-Ami et al., 2017). ERG11 is the most prevalent missense mutation reported in drug resistance genes, and that Y132F amino acid substitution is a common mechanism of azole drug resistance (Ahmad et al., 2020; Chow et al., 2020). While Ahmad et al. reported that Y132F-containing strains were susceptible to VOR, healeyet al. discovered that Y132F-containing isolates exhibited cross-resistance to VOR (Healey et al., 2018; Ahmad et al., 2020). In this investigation, we discovered the contrary result: VOR resistance exists, and the different drug sensitivity test methods may cause this different result.

CDR1 belongs to the efflux pump transporter gene, and the over expression of CDR1 of *C.aureus* causes azole drug resistance, when the CDR1 gene is absent, the MIC values of itraconazole and fluconazole can be lowered by 64-128 times (Rybak et al., 2019). Mohammad et al. Reported that among the strains resistant to azoles is candida near smooth; 12 of the 22 strains resistant to azoles were found by WGS and targeted plasmid sequencing and all of them had a novel mutant gene site CDR1-N1132D that we also included in this study sample (Asadzadeh et al., 2021). TAC1B is a zinc family transcription factor that has been shown to be altered in the coding genes of a substantial number of fluconazole-resistant strains (Rybak et al., 2020). Simultaneously, introducing the fluconazole-resistant gene, including TAC1B, into fluconazole-sensitive strains doubled the MIC value of fluconazole, and the MIC value of fluconazole was adjusted. Fluconazole’s MIC value has decreased, demonstrating that the TAC1B mutation affects fluconazole resistance. TAC1B-A583 gene locus has been identified in previous literature. The Q503E mutation site has not previously been published, and novel mutation sites were discovered during this investigation.

In addition to the Q503E, CDR1-E709D, and TAC1B-Q503E are unique mutation sites in this isolated sample that differ from drug-resistance gene sites described in other places and may be associated with various geographical areas.

This strain is comparable to the strain discovered in China by Professor Wang Hui (Wang et al., 2018). It causes only local urinary symptoms and is resistant to conventional antifungal agents, particularly azoles such as fluconazole, but is generally susceptible to echinocandins. In addition to urinary tract infections, other infections of the body can also be treated. Current *C. auris* isolates are sensitive to amphotericin B and echinocandin. This case demonstrates that the elderly have underlying diseases, impaired consciousness, physical impairment, tracheal intubation, and other diseases and that individuals with low immunity are more susceptible to infection. *Candida albicans* was cultured in urine prior to isolating the *C. auris* strain, and antifungal medications were administered. This is consistent with reports that the detection of *C. auris* is linked to the detection of other Candida species in the background and the use of antifungal medications. Over the past five years, China has reported the isolation of over 100 *C. auris* strains from 60 patients. These strains exhibit varying geographic and phylogenetic characteristics, genetic clades, mating types, and mutations associated with antifungal resistance across diverse clinical scenarios (Du et al., 2022).

### 2.3 Literature review

Since 2009, when *C. auris* was identified as a novel species, it has been increasingly reported as a source of invasive opportunistic illnesses. With the first cases being reported in Europe and the United States in 2016, surveillance organizations issued alerts to be on the lookout for this fast growing and new infection. We present a case of *C. auris* infection that occurred in a hospitalized patient. Our example is illustrative of atypical *C. auris* resistance compared to more common clinical yeasts: its potential for health-care-associated spread from endemic areas, its potential for acquired multidrug resistance, and the identification difficulties it poses. We provide our clinical case alongside a brief literature review.

### 2.4 Epidemiology

*C. auris* was first reported as a novel yeast species in East Asia, but its clinical importance was not totally obvious. Ear samples taken from individuals suffering from chronic otitis media were where surveillance studies first discovered *C. auris*. It was unknown if it had a pathogenic role in these situations (Kim et al., 2009; Satoh et al., 2009). Because of the inconsistency in the findings that biochemical identification techniques produced, this group of isolates garnered attention. The sequences of the isolate’s 26S rDNA D1/D2 domain and ITS region exhibited only 85.7% and 87.5% similarity to *C. haemulonii*, respectively, when compared to those of *C. haemulonii*. After further investigation, it was shown that the Candida isolate had a phylogenetic relationship with *Candida pseudohaemulonii, Candida heveicola*, and *Candida ruelliae*. In addition, the biochemical analysis of the isolate demonstrated distinct characteristics from those exhibited by other species of Candida, such as distinct patterns of carbon assimilation and the capacity to grow at a temperature of 42 degrees Celsius. These findings provided additional confirmation that *C. auris* is a novel Candida species (Hata et al., 2019). In addition, examination of 15,271 Candida isolates collected from Asia, Latin America, Europe, and North America indicated the existence of four *C. auris* isolates collected between 2009 and 2015, providing more evidence for the notion that *C. auris* is an emerging human disease (Forsberg et al., 2018). The whole genomes of 47 *C. auris* isolates were sequenced using whole-genome sequencing (WGS). These isolates were acquired from a variety of geographic areas (i.e., India, Pakistan, Japan, Venezuela, and South Africa). The examination of the entire genome sequences using single-nucleotide polymorphism showed four clades that are geographically different from one another. These lineages include Clade I (South Asian), Clade II (East Asian), Clade III (African), and Clade IV (South American). The changes in single nucleotide polymorphisms, or SNPs, that were discovered between the samples from each branch provide evidence that the organism has undergone rapid evolution (Lockhart et al., 2016). However, within their geographic group, the isolates had very little genetic differences among themselves (Welsh et al., 2017). Iran has been identified as the location of the recently developed fifth *C. auris* clade, which differs from previous clades by more than 200,000 SNPs (Chow et al., 2019). It is interesting to note that Clade II has been recorded to produce ear infections, despite the fact that Clade I, III, and IV were primarily responsible for nosocomial transmission and large-scale epidemics in healthcare facilities; yet, Clade II did not cause invasive infections (Welsh et al., 2019).

*C. auris* was identified as the causative agent in three instances of bloodstream infection in 2011, according to the findings of a multicenter candidemia monitoring study conducted in South Korea (Lee et al., 2011). One of the isolates was from a catheter-related infection that occurred in 1996 and included a yeast that had not been detected before. India was the location where the first cases of *C. auris* fungemia were reported (2014) (Chowdhary et al., 2013), Kuwait and South Africa (2014) (Magobo et al., 2014; Emara et al., 2015), and from the Americas and Europe in 2016 (Calvo et al., 2016; Schelenz et al., 2016). In retrospect, instances of *C. auris* infection that were either unreported or unidentified had previously happened in South Korea in the middle of the 2000s, and they had occurred in India in 2009 (Kim et al., 2009; Chowdhary et al., 2013). Presently *C. auris* is an established cause of nosocomial yeast infections in many countries worldwide. In Kuwait, over 50 cases have occurred, all involving critically ill patients (Khan et al., 2018). Continental Europe’s first outbreak occurred in 2016 in a Spanish tertiary center. It affected 140 patients (including 41 candidemias) during its first year. Despite early implementation of strict measures, the outbreak acquired an endemic pattern, with newly colonized patients being persistently identified over 2 years after the first cases (Ruiz-Gaitán et al., 2018). We were able to discover that this particular instance is one of an increasing number of *C. auris* infections that are progressively happening not just in hospitals in China but also throughout the globe. Its exceptional capacity for clonal propagation and rapid metamorphosis from an unknown species into a significant pathogen are two unusual elements of its epidemiology that stand out in comparison to those of other clinical yeasts. The metamorphosis is still poorly understood. It was shown that its appearance as a clinical pathogen occurred just very recently. The concept that *C. auris* was already widely circulating before it was subsequently designated as a distinct species has been debunked by retrospective analyses of yeast stock collections. The biggest research, which was a retrospective analysis, looked at 15,271 candidemia isolates collected from medical institutions throughout Asia, Europe, South America, and North America between the years 2004 and 2015 (Lockhart et al., 2017). Only one specimen that had been incorrectly diagnosed as *C. haemulonii* was later found to be *C. auris*; the isolate in question dated all the way back to 2009. One Pakistani isolate from 2008 was successfully reidentified as *C. auris* after being transferred to a different population. Korea is the location where the first known strains of *C. auris* have been reported. In addition to the strain that was isolated in 1996 by Lee et al. (2011), it is now known that 15 strains that were collected between the years 2004 and 2006 are also *C. auris* (Kim et al., 2009). For instance, in Kuwait, all uncommon clinical Candida strains collected beginning in 2005 were sequenced using the ITS method, but no *C. auris* isolates were detected until the year 2014 (Khan et al., 2018). These numbers provide credence to the hypothesis that the advent of *C. auris* around the globe has just occurred during the last few decades, and that this species was not only neglected or misidentified (Lockhart et al., 2017).

### 2.5 Predisposing factors

The majority of clinical studies conducted in Asia have concluded that immunocompromised patients, medical comorbidities or underlying chronic diseases, the use of central venous and urinary catheters, extended stays in intensive care units, and previous exposure to a variety of antimicrobials are the primary risk factors for *C. auris* infection (Chakrabarti et al., 2015; Rudramurthy et al., 2017; Caceres et al., 2019). Indian research found that almost all of the patients with candidemia in the intensive care unit (11 out of 12) were immunosuppressed as a result of underlying chronic conditions and had lengthy stays (Chowdhary et al., 2013). The identified risk factors had a strong correlation with a *C. auris* outbreak in Venezuela, in which all cases had previous exposure to antibiotics and had undergone invasive medical interventions prior to acquiring *C. auris* candidemia. In addition, all of the cases had been diagnosed with *C. auris* candidemia (Calvo et al., 2016).

## 3 Diagnosis

Infections caused by *C. auris* may be identified in conventional microbiology labs by the process of cultivating samples of bodily fluids, blood, or specimens from the afflicted locations (Sarma and Upadhyay, 2017).

The accuracy of the fungal pathogen diagnosis is still dependent on the precision with which *C. auris* colonies are picked from primary culture plates, regardless of the diagnostic procedures that are used. At this time, the Salt Sabouraud Dulcitol enrichment broth methodology that was first presented by Welsh et al. (2017) is being used in order to isolate *C. auris* from specimens that have been acquired from clinical and environmental sources (Clark et al., 2013). Isolates from South Korea and Japan were originally included in the library databases of MALDI-TOF MS systems (the Bruker-Daltonics MALDI Biotyper and the bioMerieux VITEK MS, respectively). Because of the growing number of newly reported strains of C. auris, it is essential to regularly update the database in order to enhance *C. auris* identification (Clancy and Nguyen, 2016). At this time, clinical labs make use of MALDI-TOF MS as a method of doing quick diagnostic testing. All of the phylogenetic clades of Candida species have been included in the latest version of the reference databases, which have been updated (Bao et al., 2018; Zhu et al., 2020). The sample preparation process for this approach of species identification is straightforward, and the turnaround time is rather quick. These are both benefits (Croxatto et al., 2012). The ITS region of *C. auris* or the D1/D2 domains of rDNA sequences have been determined, and both of these methods have allowed for the accurate and trustworthy identification of *C. auris* (Ahmad and Alfouzan, 2021). Ninan et al. brought attention to the significance of the sequencing approach in light of the fact that there is a chance that *C. auris* might be incorrectly identified in clinical labs (Ninan et al., 2020). in view of the possibility of misidentifying *C. auris* in clinical laboratories. In a nutshell, rDNA sequencing and MALDI-TOF MS have been regarded as the most reliable, rapid, and efficient approaches in the identification of *C. auris*. On the other hand, the equipment required for these methods is not available for every clinical laboratory due to the high cost and the technical demands of these methods (Kwon et al., 2019). Assays based on molecular biology may be used in clinical labs for the purpose of routine identification of fungal species since they are capable of producing trustworthy findings in a matter of a few hours (Ahmad and Alfouzan, 2021).

### 3.1 Mode of transmission and colony formation

The majority of C. aurisspecimens were retrieved from hospital settings, particularly wet surfaces. This suggests that potentially infectious surfaces in healthcare facilities might be the source of transmission (Cadnum et al., 2017; Vallabhaneni et al., 2017). According to Rossato et al., the capacity of *C. auris* to form cellular aggregates, in addition to its resistance toward high salinity and temperatures of up to 42°C, all contribute to its persistence in the hospital environment (Rossato and Colombo, 2018). Patterson et al. documented a minor *C. auris* epidemic in intensive care units (ICUs), in which the source of the *C. auris* infection was determined to be a cloth lanyard that held a key to access restricted drugs and was often used by nursing staff in two ICUs. Because the lanyard is constructed of a material similar to polyester or nylon, the yeast pathogen was able to attach to it and continue to live there for at least two weeks. *C. auris* has been isolated from items that are not considered to be medical supplies, such as curtains, flooring, windows, and bed rails (Patterson et al., 2021). However, their function in the process of transmission has not yet been proven. Previous research has shown that the presence of *C. auris* when it is isolated from non-sterile body locations is more likely indicative of colonization than it is of infection (Pappas et al., 2015; Sabino et al., 2020). Because of the continuous use of intravenous broad-spectrum antibiotics, prolonged ICU stays, excessive use of medical devices, and inadequate surveillance strategies, Das et al. observed *C. auris* colonization at various anatomical sites of the body, such as the axilla, tracheostomy, and groin. This was the result of *C. auris* colonization at different anatomical sites of the body. It is possible that colonization of these anatomical areas by *C. auris*, which may cause infection in the bloodstream, is one of the reasons causing the illness (Das et al., 2018). Colonization of *C. auris* on the skin of patients and possibly on other regions of their bodies may have aided the horizontal spread of the infection to additional patients through shedding and persistence in the healthcare environment (Tsay et al., 2017). In addition, *C. auris* is able to maintain its viability and continue to exist on abiotic surfaces such as steel and plastic for a period of many months (Short et al., 2019). According to the findings of Piedrahita and colleagues, Candida species, including *C. auris*, exhibit survival rates that are equivalent on both dry and damp surfaces (Piedrahita et al., 2017). As soon as *C. auris* colonizes a hospital setting, it activates the stress-activated protein kinase Hog1 in order to adapt to its new surroundings and maintain the cell phenotype it has acquired (Chakrabarti and Sood, 2021). *C. auris*, in a way that is dependent on the strain, will produce the enzymes phospholipase and proteinase during this transition in order to assist in its pathogenesis (Larkin et al., 2017). To begin, the hydrolytic enzymes activate the glycosylphosphatidylinositol (GPI)-anchored cell wall proteins and adhesins for the purpose of adhering of biofilms to healthcare surfaces and medical equipment via nonspecific interactions such as particular adhesin–ligand connections (Larkin et al., 2017). Phospholipases are essential to the development of the infection because they cause damage to the host cell and help the virus avoid the host immune system (Johnson et al., 2018). Second, *C. auris* is able to develop resistance to osmotic stress as well as disinfectants that are often used in healthcare facilities by producing extracellular matrix in biofilms. This occurs as a result of the expression of the KRE6 and EXG genes. As a consequence of this, it is possible for *C. auris* to infect the skin of healthcare workers as well as patients in intensive care units via direct contact with infected surfaces (Chakrabarti and Sood, 2021). The third thing that *C. auris* does is release hemolysins, which help it absorb iron from the hemoglobin–heme group in the host’s blood in order to improve its chances of surviving within the host (Furlaneto et al., 2018). It’s possible that these virulence variables have significant contributions to the fast spread of *C. auris* in healthcare facilities. The fact that *C. auris* secreted aspartyl proteinase (SAP) is more active at a temperature of 42 degrees Celsius demonstrates that the yeast pathogen is able to maintain its pathogenicity even when exposed to higher temperatures (de Jong and Hagen, 2019). In addition, the research into an epidemic that took place in Colombia uncovered a relationship between patients, healthcare staff, and the environment in regards to the spread of *C. auris*. The strains that were recovered from sick patients and also the surroundings are genetically similar to the viruses that were identified by healthcare professionals (Escandón et al., 2018). *C. auris* is less able to form biofilms than *C. albicans*, but it can form biofilms that increase antifungal drug resistance, which has been linked to infections associated with implants (Taff et al., 2013; Sherry et al., 2017). These infections include bloodstream infections, infections of the central nervous system, and infections of prosthetic joints (Horton and Nett, 2020). *C. auris* which was isolated from catheter-associated candidemia in a mouse model demonstrated adhesion and growth as yeast cell biofilms on catheter surfaces (Horton and Nett, 2020). According to Misseri et al. it is interesting to note that the appearance of *C. auris* and the disease’s ability to be transmitted to people may have a connection with global warming. The ability of *C. auris* to withstand high temperatures and high salinity may be due, at least in part, to the influence that climate change has on wetlands. It is possible that *C. auris* gained the virulence components that contribute to its persistence in the ecosystem as a result of an induced genetic mutation, which may have been caused by a combination of the impacts of global warming and UV radiation. In a nutshell, the high transmission rates among patients and healthcare workers in clinical settings may be explained by the fact that *C. auris* can live on medical equipment for a long amount of time, offers a high infectious risk, and is able to survive there for an extended period of time (Misseri et al., 2019).

### 3.2 Precautions to prevent the spread of infection

It is of the highest need to develop effective infection prevention and control (IPC) procedures and screening protocols in order to manage infection and transmission in healthcare settings, where *C. auris* is becoming more prevalent and is continuing to exist. At this time, there is no environmental cleaning or disinfection procedure that is standardized that can be used to control the nosocomial spread of *C. auris* in healthcare settings. It has been noted that quaternary ammonium compounds like Lysol and Virex II 256, which are used for disinfection in certain healthcare settings, are not effective against *C. auris* (Caceres et al., 2019). A range of cleaning or disinfection strategies, including the use of disinfectants and cleaning chemicals, have been suggested by a number of groups concerned with public health. Patients who are colonized or infected with an infectious disease should be isolated in a private room, as recommended by the CDC, and active cleaning with an effective hospital-grade disinfectant that is registered with the Environmental Protection Agency (EPA) should be required to disinfect any contaminated surfaces (Ku et al., 2018). In a similar way, Public Health England (PHE) recommends that products based on hypochlorite (1000 ppm) be used for the cleaning of environmental spaces. In the meanwhile, the European Center for Disease Prevention and Control (ECDC) suggests using disinfectants with verified antifungal action for cleaning terminals. In addition to terminal cleaning, certain health agencies also suggest frequent cleaning with a chlorine-based disinfection solution at a concentration of 1000 PPM (Ku et al., 2018). Studies conducted in the past have shown that disinfectants containing chlorine are efficient against *C. auris* (Moore et al., 2017). In addition, the efficiency of various concentrations of chlorine-based disinfectants in killing *C. auris* on surfaces such as cellulose matrix, stainless steel, and polyester during 5 and 10 minutes of contact time was shown to be comparable (Kean et al., 2018). The eradication of *C. auris* isolates by 96.6–100% was shown upon exposure to hydrogen peroxide vapor (Abdolrasouli et al., 2017).

Overall, it’s clear that stopping the spread of *C. auris* in hospitals requires strict intervention and strong preventative management measures. Patient isolation and contact tracing, healthcare workers wearing PPE and practicing good hand hygiene, culture and molecular surveillance of patients and environmental surfaces, routine and terminal cleaning of healthcare facilities, and decontamination of the skin with the appropriate disinfectants and antiseptics are all part of an effective infection control program.

## 4 Conclusion and perspective

*C. auris* is a relatively novel yeast species that has quickly become a significant global source of opportunistic invasive infections. Our case is one of a growing number of infections arising in non-endemic regions, demonstrating the yeast’s exceptional capacity for healthcare-associated transmission. In addition, our example exemplifies the problems associated with regularly used biochemical and MALDI-TOF MS identification technologies, such as misidentifications and poor identification consistency scores. Manufacturers have just lately adapted identifying systems for clinically proven and dependable usage. Multiple drug resistance in *C. auris* has garnered significant attention in recent years. The United States CDC has produced prevention and control techniques to prevent the spread of *C. auris* in medical settings. The sequencing of the whole genome exposes the drug-resistant genes, and future research will identify other mutation locations. The clinical field is the source of the isolated strains, and the manner in which they infect and propagate requires more examination. What are the reasons for the similarities of mutation sites in different geographical branches, and how does the drug resistance mechanism occur, require further investigation, scientific support for future research on novel drugs, and clinical treatment guidelines for newly discovered high-risk pathogens.

## Data availability statement

The original contributions presented in the study are included in the article/**Supplementary Material**. Further inquiries can be directed to the corresponding author.

## Ethics statement

The patient agreed to participate in the research. However, the study has not been reviewed or approved by a human research ethics committee because no harm or intervention from conducting this research will happen to participant. The studies were conducted in accordance with the local legislation and institutional requirements. Written informed consent for participation was not required from the participants or the participants’ legal guardians/next of kin in accordance with the national legislation and institutional requirements because conducting this research will not affect patient. Written informed consent was obtained from the participant/patient(s) for the publication of this case report.

## Author contributions

HH, and YX designed the research; LYi and MJ collected the data. HH, AA and LYi analyzed the data and wrote the draft. LYu, PS, HH, YY, YXW and AA revised manuscript. All authors contributed to the article and approved the submitted version.

## Supplementary material

Supplementary Figure 1 MALDI - TOF MS Identification Results.
