# Supplementary figures and images for: Candida auris infection; diagnosis, and resistance mechanism using high-throughput sequencing technology: a case report and literature review
Source: Front Cell Infect Microbiol. 2023 Dec 8;13:1211626. doi: 10.3389/fcimb.2023.1211626 (PMC10739385; doi:10.3389/fcimb.2023.1211626)

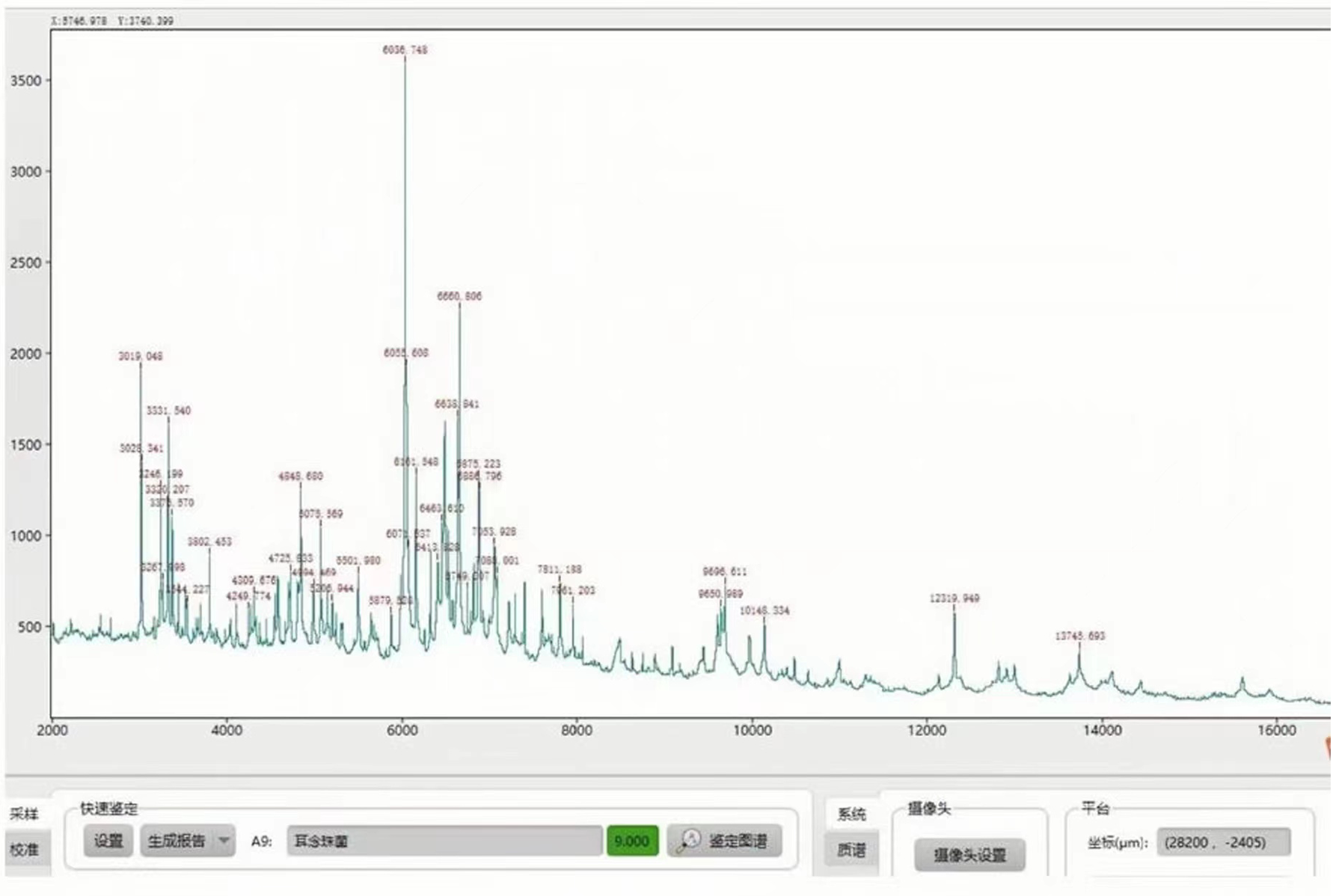

Supplement: Supplementary Figure 1 — MALDI - TOF MS Identification Results. [file Image_1.tif]
